# Kappa free light chain index in CSF diagnostics: the impact of different immunoglobulin isotypes

**DOI:** 10.3389/fimmu.2026.1747659

**Published:** 2026-02-17

**Authors:** Martin Schmidauer, Klaus Berek, Michael Auer, Franziska Di Pauli, Fabian Föttinger, Nik Krajnc, Florian Deisenhammer, Gabriel Bsteh, Janette Walde, Harald Hegen

**Affiliations:** 1 Department of Neurology, Medical University of Innsbruck, Innsbruck, Austria; 2 Department of Neurology, Medical University of Vienna, Vienna, Austria; 3 Comprehensive Centre for Clinical Neurosciences and Mental Health, Medical University of Vienna, Vienna, Austria; 4 Department of Statistics, Faculty of Economics and Statistics, University of Innsbruck, Innsbruck, Austria

**Keywords:** biomarker, crebrospinal fluid, diagnostic, IgG, IgM, Kappa (κ) FLC, multiple sclerosis

## Abstract

**Background:**

The kappa free light chain (κ-FLC) index is a sensitive marker of intrathecal immunoglobulin (Ig) synthesis and is increasingly used in cerebrospinal fluid (CSF) analysis of patients with suspected multiple sclerosis (MS). The relative contribution of the different Ig isotypes to intrathecal κ-FLC production remains unclear.

**Methods:**

We retrospectively analysed CSF data from patients with a first demyelinating event suggestive of MS enrolled in studies at the Medical Universities of Innsbruck and Vienna. Of all included patients, results on Ig and κ-FLC concentrations in CSF and serum were available. Linear regression analysis was used to assess the impact of Ig intrathecal fractions (IF) on κ-FLC index.

**Results:**

A total of 188 patients with a median age of 31 (25-39) years and a predominantly female sex distribution (62%) were included. The κ-FLC index was significantly higher in patients with isolated intrathecal IgG synthesis [32.5 (17.7-81.0); n=130] compared to patients without intrathecal immunoglobulin production [3.0 (2.0-5.9); p<0.001; n=18] and was further elevated in patients with both intrathecal IgG and IgM synthesis [68.4 (48.4-120.6); n=29]. Both IgG and IgM IF independently contributed to the κ-FLC index in linear regression analysis, with IgG IF having approximately 3.5 times the effect size of IgM IF. Exploratory analysis of the contribution of IgA IF to κ-FLC index revealed qualitatively the same results.

**Conclusion:**

Increase of κ-FLC index in patients with MS is predominantly due to an intrathecal IgG synthesis, while the contribution of intrathecal IgM is less frequent and quantitatively low.

## Introduction

The synthesis of immunoglobulins (Ig) within the intrathecal compartment is a hallmark of multiple sclerosis (MS) (1–3) and other inflammatory neurological diseases (4). Different methods are available to detect intrathecally produced Ig. These include the quantitative measurement of IgA, IgM and IgG in paired cerebrospinal fluid (CSF) and serum samples followed by the calculation of the intrathecal fraction (IF) (5, 6), as well as the qualitative detection of CSF-restricted IgG oligoclonal bands (OCB) (7). In recent years, the quantification of kappa free light chains (κ-FLC) in CSF and serum followed by the calculation of the κ-FLC index has been introduced into routine CSF diagnostics (8). κ-FLC are produced by plasma cells in excess to intact immunoglobulins and also accumulate in the intrathecal compartment during inflammatory diseases of the central nervous system (CNS) (9).

While determination of the Ig IF shows only moderate diagnostic sensitivity (4), the detection of CSF-restricted OCB is technically demanding, time-consuming and exclusively reflects intrathecal IgG synthesis. The κ-FLC index overcomes the weaknesses of these two approaches. Measurement of κ-FLC is easy, fast, cost-effective and reliable (9) and the κ-FLC index shows a high diagnostic sensitivity and specificity comparable to OCB (10). Furthermore, the κ-FLC index increases not only in case of intrathecal IgG but also in case of intrathecal IgM and/or IgA synthesis (11, 12).

However, the relative contribution of intrathecal synthesis of the different Ig isotypes, i.e., of IgG, IgA and IgM, to an intrathecal κ-FLC synthesis, i.e., to an increase of κ-FLC index, in patients with MS is not known, which is why we performed the present study.

## Methods

### Patients and samples

We included patients from observational studies at the Medical University of Innsbruck [Risk Assessment in Multiple Sclerosis by Cerebrospinal Fluid Free Light Chains (RIMSC) (13) and Biomarker Risk Assessment in Multiple Sclerosis (BRAMS)] and the Medical University of Vienna [Austrian Multiple Sclerosis Cohort (AMSC)] (14). For this study, in patients with a first demyelinating event of the CNS suggestive of MS, lumbar puncture (LP) and determination of Ig IF as well as of the κ-FLC index were necessary for inclusion. All patients were treatment-naïve at the time of LP. Details on inclusion of patients are given in **Supplementary Figure S1**. Diagnosis of MS was made using the 2017 revised McDonald criteria (1).

All CSF samples were collected by LP. Serum samples were collected concomitantly within 30 minutes via venipuncture. Samples were centrifuged at 2000 g for 10 minutes at room temperature before measurement (15).

### Routine cerebrospinal fluid analysis

White blood cell (WBC) and red blood cell (RBC) counts were determined using Fuchs-Rosenthal chamber (MUI) (4), or automated by Sysmex confirmed by visual counting using Fuchs-Rosenthal chamber if counts were ≥5/μL (MUV).

Immunoglobulin concentrations were determined by nephelometry. Intrathecal synthesis of IgG, IgM and IgA was calculated using the Auer & Hegen formulae as previously published (5). Detection of OCB was performed by isoelectric focusing and subsequent immunoblotting using IgG-specific antibody staining at both centres as previously described (7).

### Determination of albumin and k-free light chains

Albumin and κ-FLC in CSF and serum were measured by nephelometry (Atellica; Siemens, Erlangen, Germany) using the N Albumin and N Latex FLC kappa assay (16, 17), respectively, according to the manufacturer’s instructions.

### Calculation of the κ-FLC Index

The κ-FLC index was calculated using the following formula:


$$k-FLC\;index\;=\;\frac{k\;-\;FLC_{CSF}\;/\;k-FLC_{Serum}}{Albumin_{CSF}\;/\;Albumin_{Serum}}$$

A κ-FLC index ≥6.1 was considered positive (10).

### Statistical analysis

Categorical variables were expressed as frequencies and percentages, and continuous variables as median, 25^th^, 75^th^ percentile and range as appropriate. For group comparisons, the Mann-Whitney-U test and Kruskal-Wallis test were applied. Bonferroni correction was done for multiple testing. Pearson correlation coefficient (r) was used for correlation analysis. Linear regression model was performed to evaluate the influence of the IF of the different Ig isotypes (continuous) on the κ-FLC index. The distribution of the κ-FLC index is highly right-skewed (**Supplementary Figure S2**). Furthermore, the impact of change of IgG IF (or IgM IF) on the κ-FLC index is not constant over the full range of possible κ-FLC index values (**Supplementary Figure S3**). Therefore, a log-transformation of k-FLC index is appropriate in linear regression.

A p-value <0.05 was considered statistically significant. In regression analyses, according to the clear one-sided hypotheses, i.e. higher κ-FLC index in case of intrathecal IgG, IgA and/or IgM synthesis (12, 13), one-sided hypothesis testing was used and, thus, one-sided p-values are shown. The unidirectional relationship between κ-FLC and Ig is based on a clear biological mechanism: Plasma cells produce intact Ig and in excess κ-FLC, i.e., in case of intrathecal plasma cell activity both Ig and κ-FLC accumulate in the intrathecal compartment (9). Therefore, the parameters that quantitatively capture intrathecal Ig and κ-FLC synthesis, i.e., IF Ig (4) as well as κ-FLC index (2, 3), increase in case intrathecal plasma cell activity.

All statistical analyses were performed in R (18).

### Ethics statement

The study was approved by the Ethics committees of the Medical Universities of Innsbruck (approval number: 1050/2023 and 1244/2019) and Vienna (approval number: 1368/2023). Informed consent was obtained from all participants. We adhered to the declaration of Helsinki and national regulations during all study procedures.

## Results

A total of 188 patients at a median age of 31 (25–39) years showing a female predominance (62%) were included into this study. One hundred seventy-seven (94%) patients were diagnosed as relapsing-remitting MS (RRMS), while the remaining patients were considered as clinically isolated syndrome. The κ-FLC index ranged from 1.3 to 508.8 and was elevated in 167 (89%) of patients. While 18 (10%) patients had no intrathecal IgG synthesis (OCB negative), 130 (69%) had isolated intrathecal IgG synthesis (OCB positive) and 29 (15%) patients had intrathecal IgG and IgM synthesis. Only 2 (1%) patients had intrathecal IgG and IgA synthesis. Further details on demographics, clinical characteristics and CSF findings are shown in **Table 1**.

**Table 1:** Demographics, clinical characteristics and CSF findings.

Demographics	
Age (years)	31 (25–39)
Sex (female)	116 (62)
Clinical characteristics	
RRMS (according 2017 McDonald criteria)	177 (94)
Clinically isolated syndrome	11 (6)
CSF findings	
WBC (/μL)	7 (3-13)
RBC (/μL)	0 (0-1)
Total protein (mg/dL)	39 (32-52)
CSF albumin (mg/dL)	19.2 (15.6-27.3)
Serum albumin (mg/dL)	4280 (4000-4520)
Q_alb_ (×10^-3^)	4.7 (3.6-6.3)
IgG IF (%) >0	112 (59.6)
IgG IF (%)*	28.9 (13.9-48.9)
IgM IF (%) >0	38 (20.2)
IgM IF (%)*	37.9 (23.6-61.3)
IgA IF (%) >0	11 (5.9)
IgA IF (%)*	8.8 (4.7-41.8)
OCB positive, n (%)	170 (90.4)
CSF κ-FLC (mg/dL)	0.2 (0.1-0.5)
Serum κ-FLC (mg/dL)	1.2 (0.9-1.5)
Q_κ-FLC_ (×10^-3^)	17.6 (7.8-40.5)
κ-FLC index	35.8 (15.2-89.2)
κ-FLC index ≥ 6.1	167 (88.8)

Data are given as median (25^th^-75^th^ percentile) and n (%), as appropriate. * Given only for patients with Ig IF >0%.

WBC, white blood count; RBC, red blood cell count; CSF, cerebrospinal fluid; Q_alb_, CSF/serum albumin ratio; Ig, immunoglobulin; IF, intrathecal fraction; RRMS, relapsing-remitting multiple sclerosis; OCB, oligoclonal bands; FLC, free light chain; Q_κ-FLC_, CSF/serum κ-FLC ratio.

**Figure 1:**
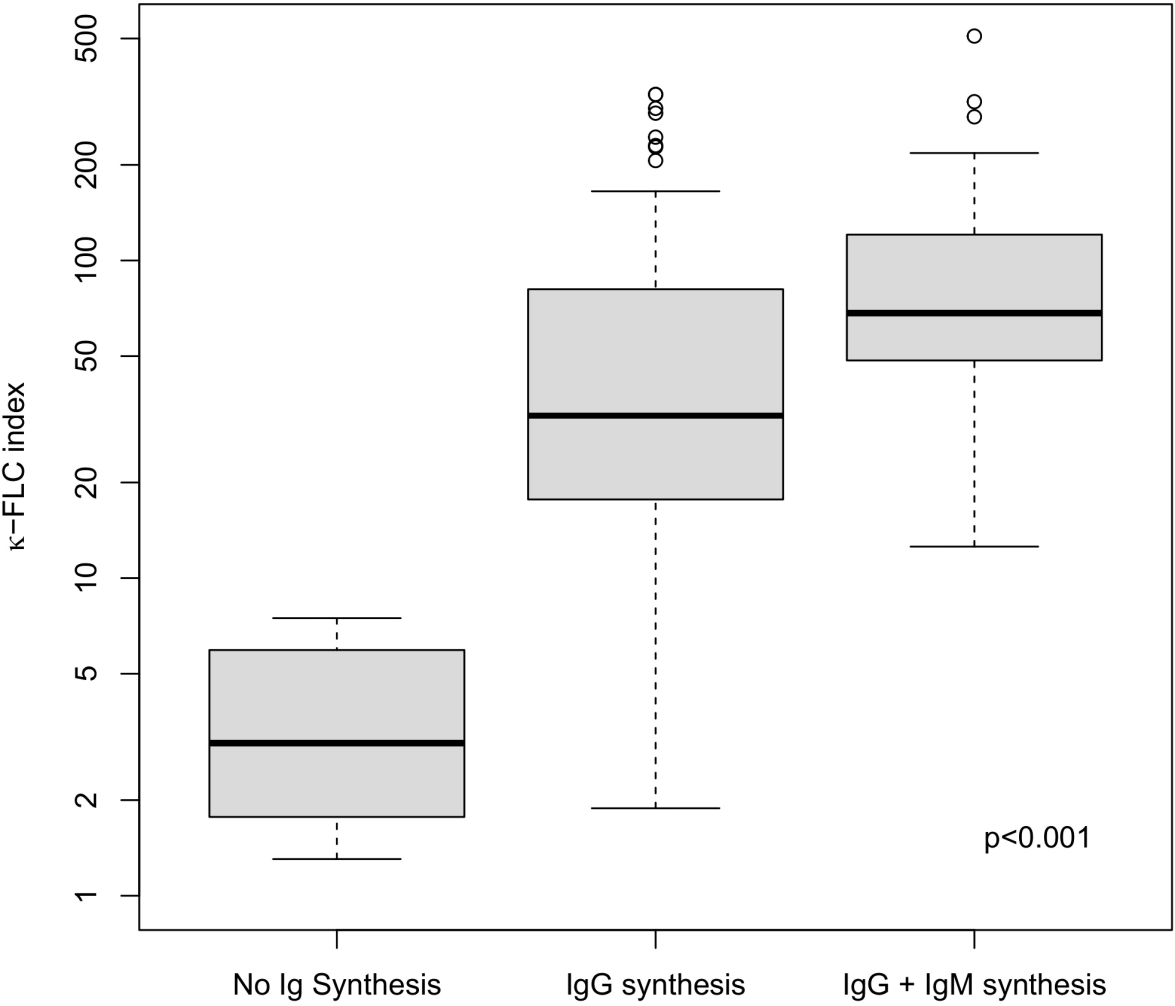
κ-FLC index according to the presence of intrathecal synthesis of different immunoglobulin isotypes. No Ig synthesis = OCB negativity and IgA IF ≤0 and IgM IF ≤0. IgG synthesis = OCB positivity. IgG + IgM synthesis = OCB positivity and IgM IF >0%. FLC, free light chain; Ig, immunoglobulin.

The κ-FLC index was statistically significantly higher in patients with isolated intrathecal IgG synthesis [32.5 (17.7-81.0)] as well as in patients with intrathecal IgG and IgM synthesis [68.4 (48.4-120.6] compared to patients with no intrathecal Ig synthesis [3.0 (2.0-5.9), both p<0.001, **Figure 1**]. The percentage IF of IgG was higher in patients with IgG and IgM synthesis compared to patients with isolated IgG synthesis (**Supplementary Figure S4**). Regarding the impact of intrathecal IgA synthesis, we refrained from group-wise statistical comparisons due to the small number of patients. A descriptive depiction is given in **Supplementary Figures S5**, **S6**. Qualitatively, the findings were similar.

**Figure 2:**
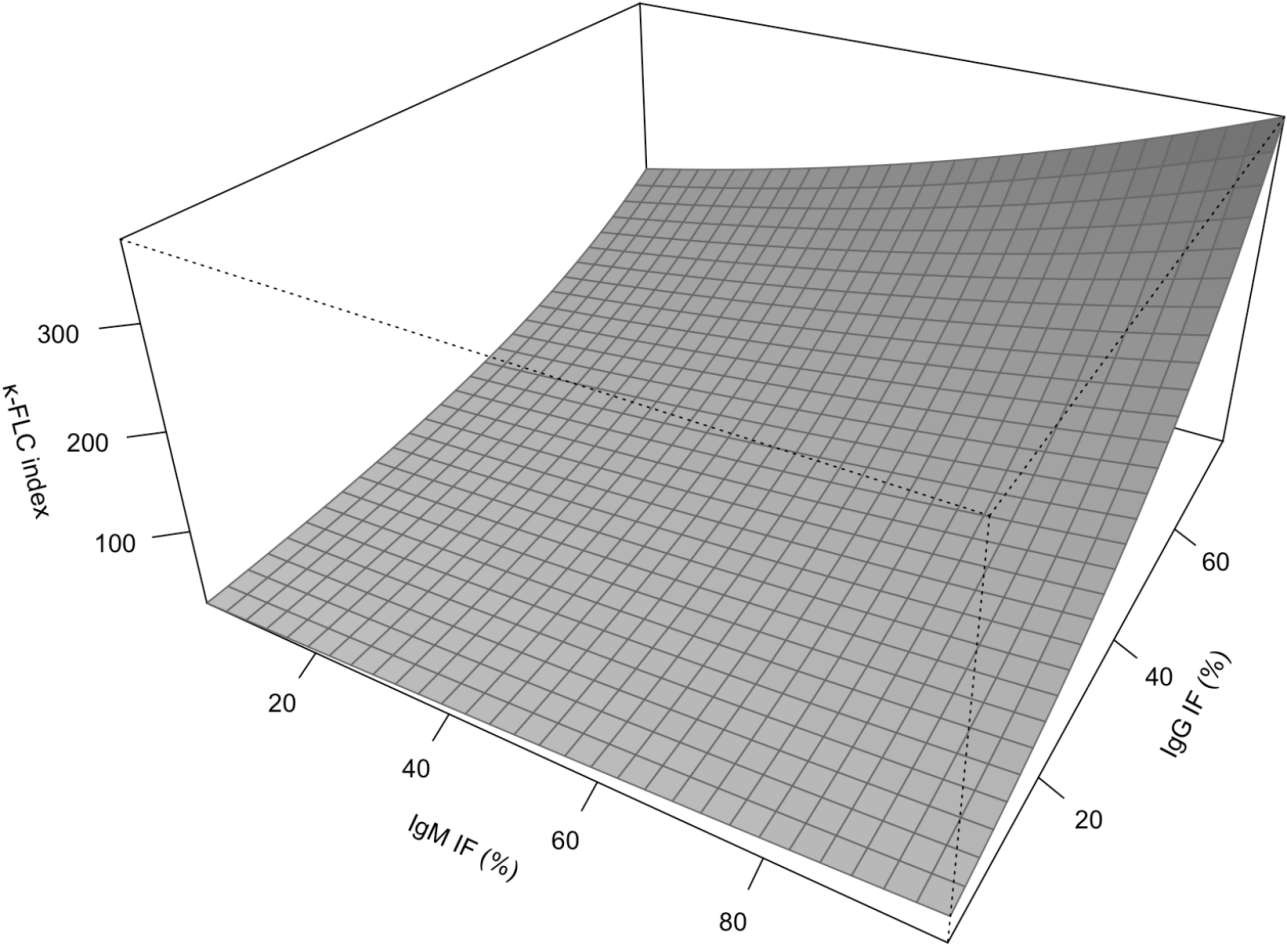
Contribution of intrathecal IgG and IgM synthesis to κ-FLC index according to multivariable model. FLC, free light chain; Ig, immunoglobulin; IF, intrathecal fraction.

Multivariable linear regression was performed to identify the relative independent contribution of intrathecal IgG synthesis and IgM synthesis to elevation of the κ-FLC index. Overall, the κ-FLC index (ln) increased with the % IgG IF (β=0.041, p<0.001) and with the % IgM IF (β=0.005, p=0.051, **Table 2A**, **Supplementary Table S1**). Similarly, in the subgroup of patients with both positive IgG as well as IgM IF, linear regression revealed qualitatively the same results, i.e. the κ-FLC index (ln) increased with % IgG IF (β=0.026, p<0.001) and % IgM IF (β=0.008, p=0.023) (**Table 2B**; **Figure 2**; **Supplementary Table S1**). Furthermore, there was no correlation between IF IgG and IF IgM (**Supplementary Figure S7**).

Table 2Multivariable linear regression analyses identifying the contribution of intrathecal IgG and IgM synthesis to the increase of κ-FLC index.

**Table:** 

(A)				
ln (κ-FLC index)	Estimate	Standard error	P-value	
			One-sided	Two-sided
IgG IF (per % increase)	0.041	0.003	<0.001	<0.001
IgM IF (per % increase)	0.005	0.003	0.051	0.101

R^2^ = 0.586, VIF = 1.1.

**Table:** 

(B)				
ln (κ-FLC index)	Estimate	Standard error	P-value	
			One-sided	Two-sided
IgG IF (per % increase)	0.026	0.004	<0.001	<0.001
IgM IF (per % increase)	0.008	0.004	0.023	0.045

R^2^ = 0.593, VIF = 1.0.

Linear regression analyses to evaluate the influence of the IgG and IgM IF on κ-FLC index using (A) the whole patient cohort and (B) the subgroup of patients positive for both IgG and IgM IF (>0%).

Due to one-sided hypothesis testing, one-sided p-values are given.

FLC, free light chain; Ig, immunoglobulin; IF, intrathecal fraction.

## Discussion

Here, we investigated to what extent the intrathecal synthesis of different immunoglobulin isotypes contributes to an intrathecal κ-FLC synthesis. We made two main observations: in patients with a first demyelinating event suggestive of MS, i) intrathecal IgG synthesis was most frequently observed (OCB: 90%, elevated IgG IF: 60% compared to elevated IgA IF and IgM IF in 6% and 20%, respectively), ii) quantitatively, intrathecal IgG synthesis is the main contributor to an increase in κ-FLC index, while the contribution of intrathecal IgM synthesis (by approximately 3.5-fold) is lower.

Plasma cells secrete intact immunoglobulins, along with excess light chains which circulate freely in body fluids including CSF (8–10). The production of FLC is not isotype-specific, that is, it is not possible to determine whether a given kappa light chain derives from an IgG-, IgA-, or IgM-producing plasma cell (19). To understand the contribution of specific immunoglobulin isotypes to κ-FLC index, it was necessary first to quantitatively assess the intrathecal production of individual immunoglobulin isotypes, and by calculating the IF of IgG, IgA and IgM (5) and correlating them with κ-FLC index values, conclusions can be made about each isotype’s relative contribution to κ-FLC index.

It is well established that intrathecal IgG and IgM production plays a significant role in MS pathophysiology (20). The detection of clonal IgG expansion in CNS – as measured qualitatively by OCB - has been a part of MS diagnostic criteria since 1983 (21). Given the high concordance and near-interchangeability of OCB detection and the κ-FLC index in MS diagnostics (8, 10), it is expected that intrathecal IgG synthesis is the predominant contributor to elevated κ-FLC index values.

With regard to IgM, previous studies highlighted a role for intrathecal IgM synthesis in MS, as it was linked to unfavourable disease course (22–24). In line with previous findings (25) we observed intrathecal IgM synthesis in 20% of patients, however, we also observed that IgM synthesis is quantitively less important. Given that the κ-FLC index holds prognostic value for MS disease course, predicting time to relapse (11), new brain MRI activity (26) and cognitive deterioration (27), it seems that the amount of general immune activation is of importance rather than a specific Ig isotype.

Intrathecal IgA synthesis was only present in 6% of patients. Due to the small number of patients with an intrathecal IgA synthesis, we could not reliably assess the relative contribution to κ-FLC index. It is known that an isolated intrathecal IgA synthesis can lead to increase in κ-FLC index (11). In MS, however, it occurs infrequently, confirming previous findings (approximately 10%) (25) and its involvement in MS is controversial and poorly understood (28). Nevertheless, we observed qualitatively the same pattern as with IgM, i.e. in case of an additional intrathecal IgA synthesis, there was also a stronger intrathecal IgG synthesis, thus, probably explaining the higher κ-FLC index. Again, we have to clearly state that role of IgA could not be confirmed in this study, as we did not have enough statistical power to reach statistical significance in the analyses.

There are some limitations of the study. First, it was a retrospective analysis with all its inherent restrictions. Second, the sample size is relatively small. Third, we included only patients with a CNS demyelinating event suggestive of MS. While this is a strength in terms of studying treatment-naïve patients (e.g., as a potential confounding effect of immune treatment on κ-FLC index levels (29) are eliminated), the contribution of different Ig isotypes to the κ-FLC index might shift in later, more chronic stages of the disease. Furthermore, our findings cannot be generalized to other inflammatory neurological diseases. This is subject to further research.

In summary, our study demonstrates that intrathecal IgG synthesis is the main contributor to intrathecal κ-FLC synthesis, i.e. increased κ-FLC index values, in patients with MS. This further substantiates the high agreement between κ-FLC index and OCB (30, 31).

## Data Availability

The raw data supporting the conclusions of this article will be made available by the authors, without undue reservation.
