# Supplementary material for: Kappa free light chain index in CSF diagnostics: the impact of different immunoglobulin isotypes
Source: Front Immunol. 2026 Feb 17;17:1747659. doi: 10.3389/fimmu.2026.1747659 (PMC12953090; doi:10.3389/fimmu.2026.1747659)
Supplement: Supplementary file 1 [file DataSheet1.pdf]

Figure S1: Inclusion of patients

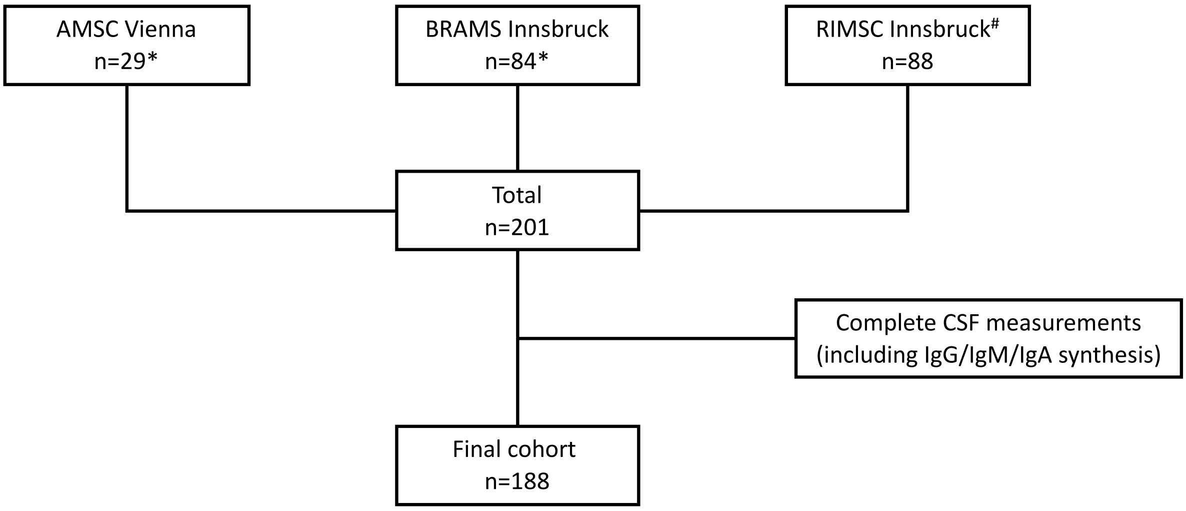

Legend:

*data extracted from database 07/25

#Berek. et. al. Neurol Neuroimmunology & Neuroinflammation, 2021 (13)

Abbreviations: AMSC, Austrian Multiple Sclerosis Cohort; BRAMS, Biomarker Risk Assessment in Multiple Sclerosis; RIMSC, Risk Assessment in Multiple Sclerosis by Cerebrospinal Fluid Free Light Chains; CSF, cerebrospinal fluid; Ig, Immunoglobulin

Figure S2: Distribution of κ-FLC index

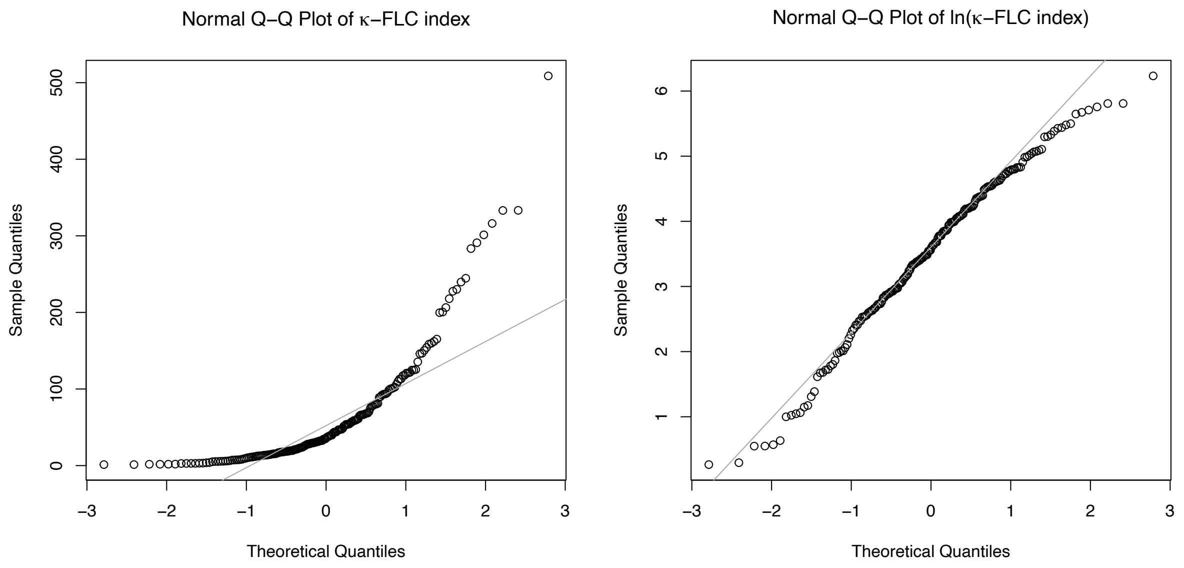

Legend:

Abbreviations: FLC, free light chain

Figure S3: Non-linear relationship between Ig IF and κ-FLC index

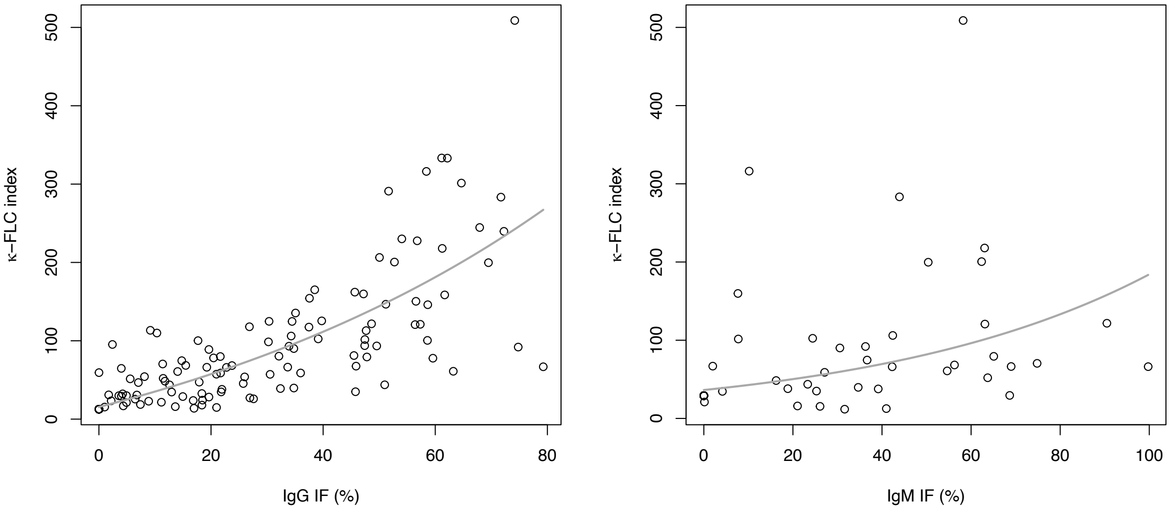

Legend:

For samples with IgG IF or IgM IF of 0%, the median of corresponding κ-FLC index values is shown.

Abbreviations: FLC, free light chain; Ig, Immunoglobulin; IF, intrathecal fraction

Figure S4: Percentage intrathecal fraction of IgG in patients with isolated IgG synthesis and in patients with combined intrathecal IgG and IgM synthesis

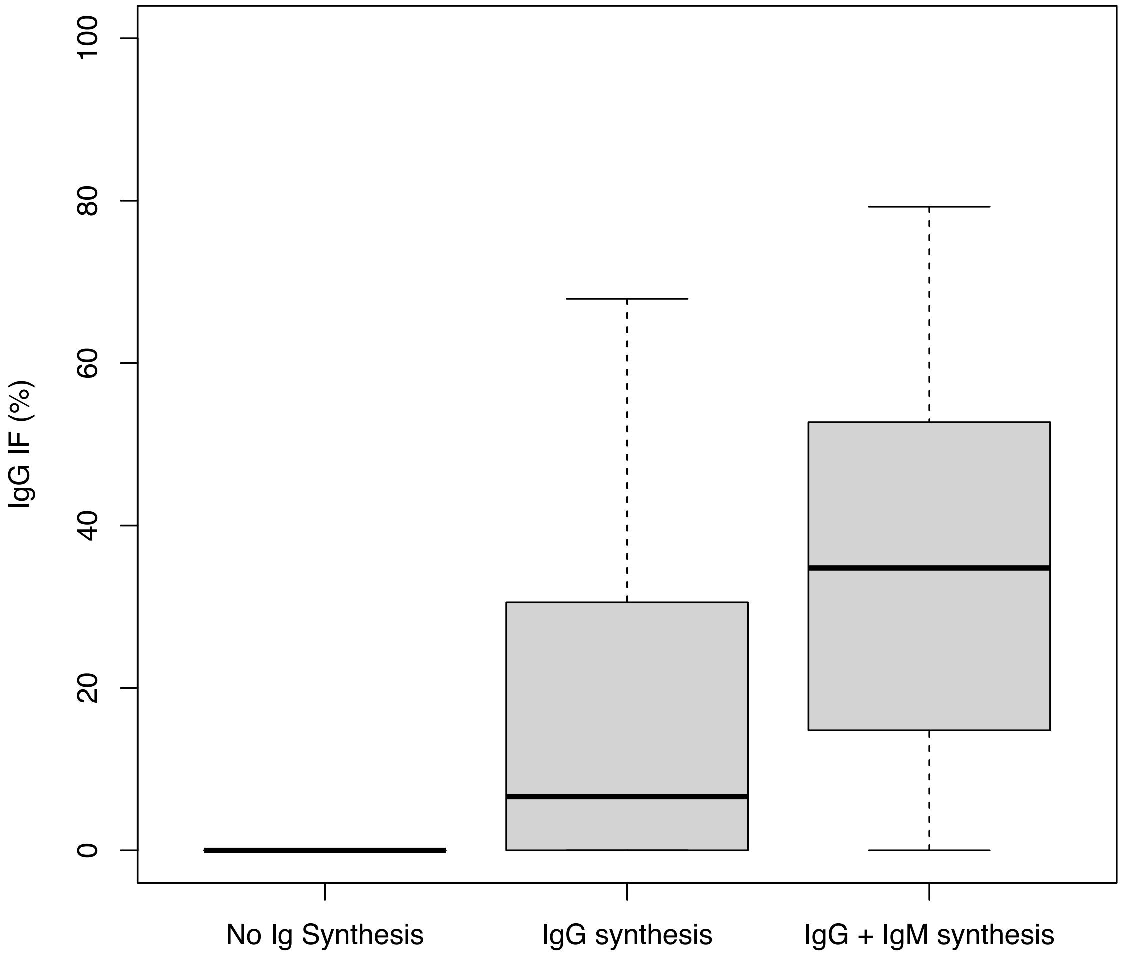

Legend:

No Ig synthesis = OCB negativity and IgA IF ≤0 and IgM IF ≤0

IgG synthesis = OCB positivity

IgG + IgM synthesis = OCB positivity and IgM IF >0%

Abbreviations: Ig, Immunoglobulin; IF, intrathecal fraction; OCB, oligoclonal bands

Figure S5: κ-FLC index according to the presence of intrathecal IgG and combined intrathecal IgG and IgA synthesis

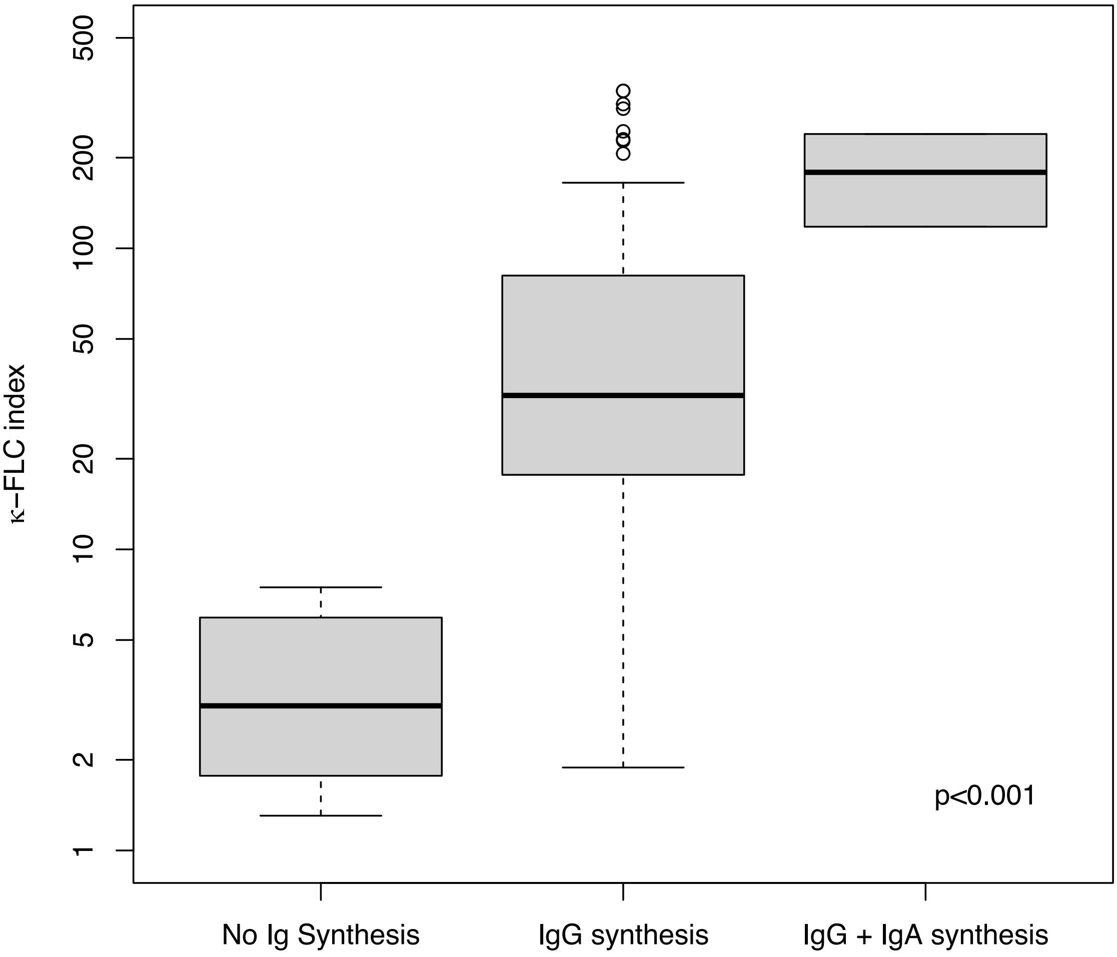

Legend:

No Ig synthesis = OCB negativity and IgA IF ≤0 and IgM IF ≤0

IgG synthesis = OCB positivity

IgG + IgA synthesis = OCB positivity and IgA IF >0%

Abbreviations: FLC, free light chain; Ig, immunoglobulin

Figure S6: Percentage intrathecal fraction of IgG in patients with isolated IgG synthesis and in patients with combined intrathecal IgG and IgA synthesis

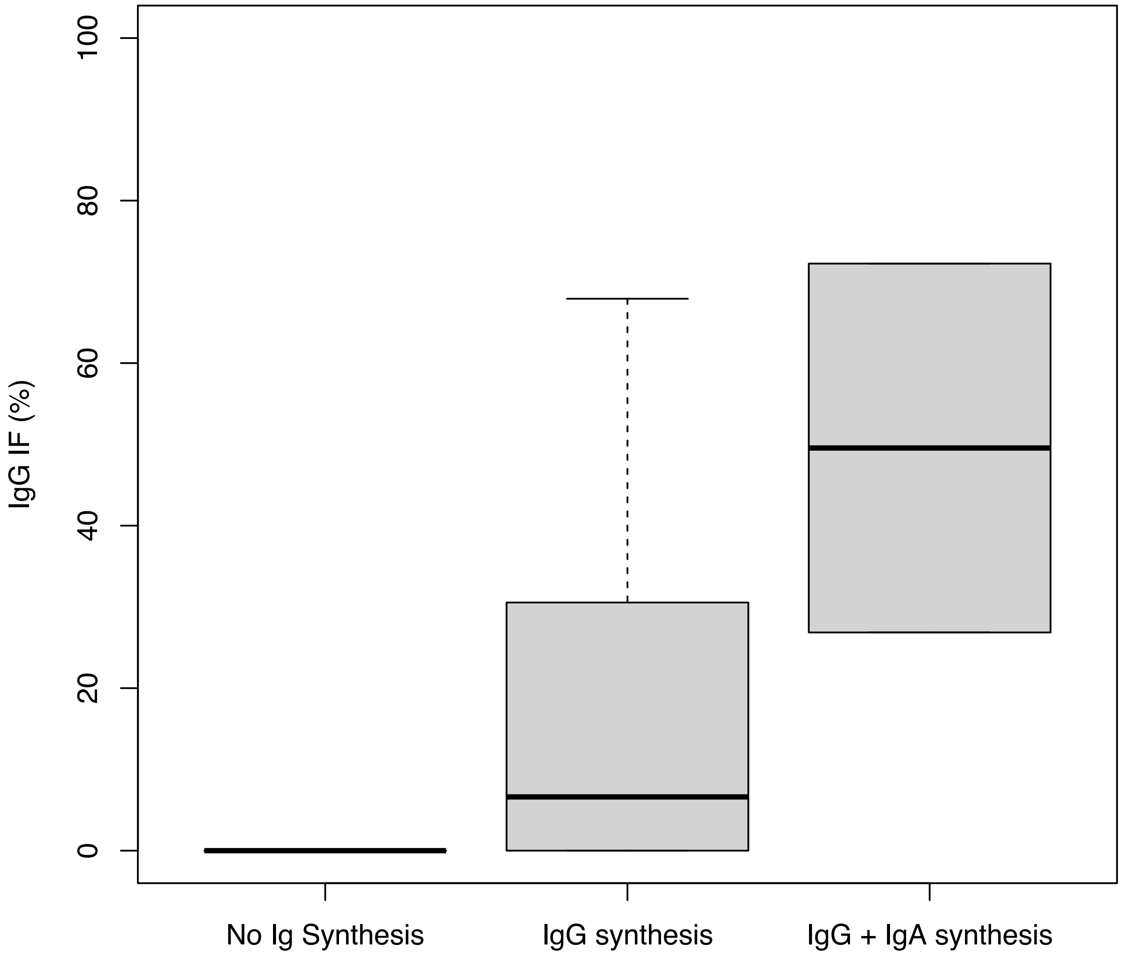

Legend:

No Ig synthesis = OCB negativity and IgA IF ≤0 and IgM IF ≤0

IgG synthesis = OCB positivity

IgG + IgA synthesis = OCB positivity and IgA IF >0%

Abbreviations: Ig, Immunoglobulin; IF, intrathecal fraction; OCB, oligoclonal bands

Figure S7: Correlation between intrathecal fractions of IgG and IgM

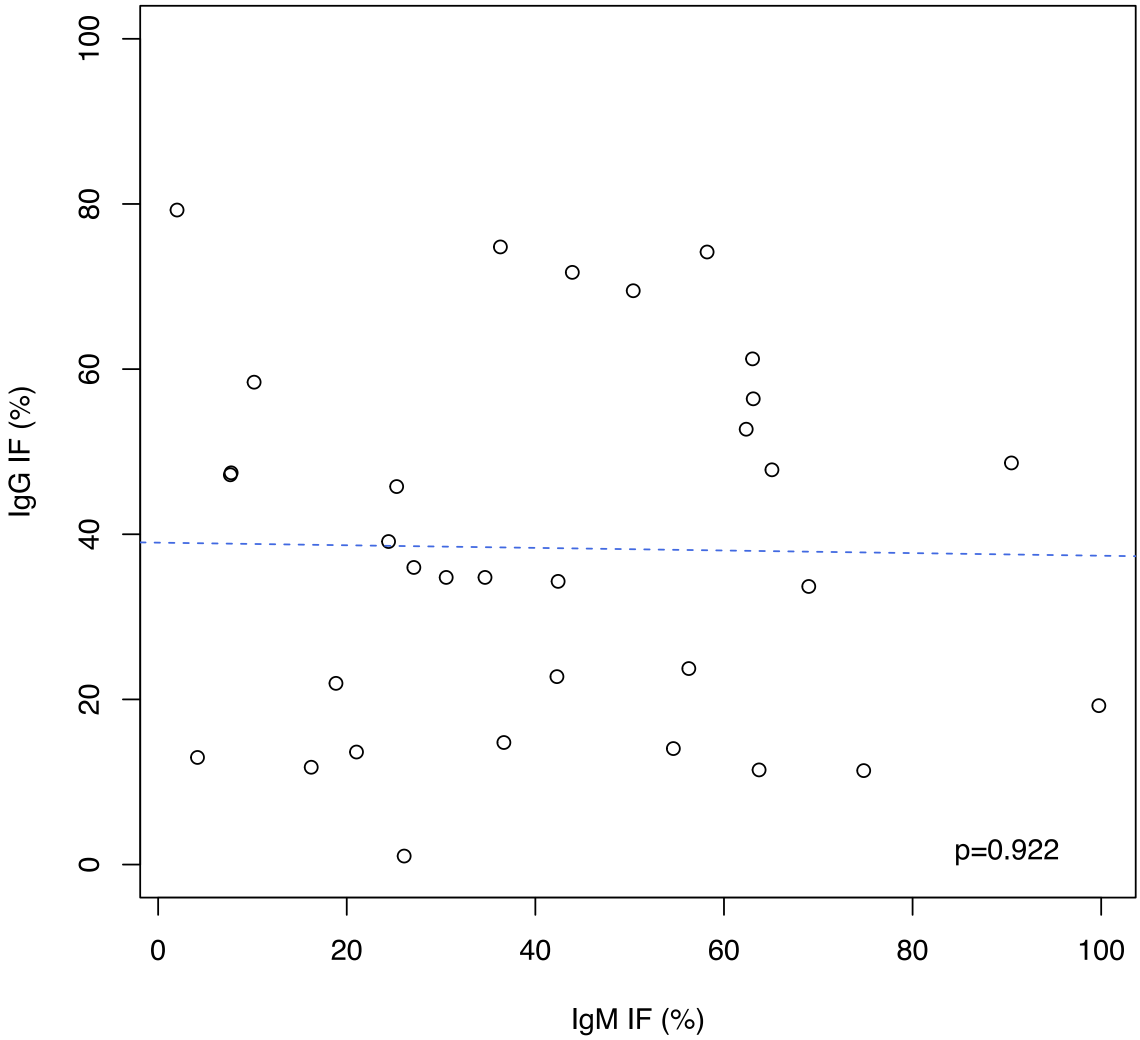

<u>Legend</u>:

Abbreviations: Ig, Immunoglobulin; IF, intrathecal fraction

Table S1: Multivariable linear regression analyses identifying the contribution of intrathecal IgG and IgM synthesis to the increase of κ-FLC index adjusted for age and sex

(A)

**ln (κ-FLC index)**	**Estimate**	**Standard error**	**P-value**
**IgG IF (per % increase)**	0.041	0.003	<0.001*
**IgM IF (per % increase)**	0.005	0.003	0.055*
**Age (years)**	-0.001	0.007	0.909
**Sex (female)**	0.169	0.126	0.180

R^2=0.590, VIF=1.1

(B)

**ln (κ-FLC index)**	**Estimate**	**Standard error**	**P-value**
**IgG IF (per % increase)**	0.026	0.004	<0.001*
**IgM IF (per % increase)**	0.008	0.004	0.025*
**Age (years)**	0.001	0.013	0.959
**Sex (female)**	0.245	0.194	0.217

R^2=0.616, VIF=1.1

Legend:

Linear regression analyses to evaluate the influence of the IgG and IgM IF on κ-FLC index using (A) the whole patient cohort and (B) the subgroup of patients positive for both IgG and IgM IF (>0%). * Due to one-sided hypothesis testing, one-sided p-values are given.

Abbreviations: FLC, free light chain; Ig, immunoglobulin; IF, intrathecal fraction
